# The Changes in Microbiotic Composition of Different Intestinal Tracts and the Effects of Supplemented *Lactobacillus* During the Formation of Goose Fatty Liver

**DOI:** 10.3389/fmicb.2022.906895

**Published:** 2022-07-18

**Authors:** Kang Wen, Long Liu, Minmeng Zhao, Tuoyu Geng, Daoqing Gong

**Affiliations:** ^1^Department of Animal Science, College of Animal Science and Technology, Yangzhou University, Yangzhou, China; ^2^Joint International Research Laboratory of Agriculture and Agri-Product Safety of the Ministry of Education of China, Yangzhou University, Yangzhou, China

**Keywords:** *foie gras*, goose, intestinal microbiota, macrogenome, *Lactobacillus*

## Abstract

Intestinal bacteria play an important role in the formation of fatty liver in animals by participating in the digestion and degradation of nutrients, producing various metabolites, and altering the barrier effect of the intestine. However, changes in the gut microbiota during the formation of goose fatty liver are unclear. In this study, 80 healthy Landes geese with similar body weights at 70 days of age were randomly divided into two groups: the control group (*n* = 48; fed *ad libitum*) and the overfeeding group (*n* = 32; overfed). The intestinal contents were collected at 0, 12, and 24 days of overfeeding. The 16S rRNA and metagenomic sequencing analyses showed that the dominant phyla were *Firmicutes, Proteobacteria, Bacteroidetes*, and *Actinobacteria*. At the genus level, *Phyllobacterium, Bacteroides, Helicobacter, Lactobacillus, Enterococcus*, and *Romboutsia* were the dominant genera in the goose intestine, and most of them were probiotics. In the control group, the relative abundance of *Firmicutes* in the jejunum and ileum gradually decreased with time, while that of *Proteobacteria* increased, whereas in the overfeeding group, the relative abundance of *Firmicutes* in the jejunum and ileum decreased and then increased with time, while that of *Proteobacteria* showed an opposite trend. In addition, supplementing *Lactobacillus* to the diet reduced body weight and fatty liver weight in overfed geese, but increased the weight of abdominal fat, suggesting that *Lactobacillus* supplementation might affect the transport of nascent fat from the liver to abdominal fat. In conclusion, the species of intestinal-dominant bacteria in the geese are relatively stable, but their relative abundance and function are affected by a number of factors. Overfeeding promotes the metabolism of nutrients in the jejunum and ileum and increases bacterial adaptability to environmental changes by enhancing their ability to process environmental and genetic information more efficiently. These findings suggest that the effect of overfeeding on the composition of intestinal microbiota may indirectly influence the formation of goose fatty liver through the gut/liver axis.

## Introduction

Goose fatty liver (or *foie gras*), an important waterfowl product with a delicious taste and flavor, is rich in unsaturated fatty acids and lecithin. Goose fatty liver is produced by 3–4 weeks of artificial overfeeding with a corn-based (>98%) diet and usually weighs 800–1,200 g, ~8–10 times the normal liver weight. The fat content of the goose fatty liver can exceed 60% (Hermier et al., 1994). Despite severe steatosis, goose livers do not develop obvious pathological symptoms, such as inflammation and fibrosis, unlike non-alcoholic fatty liver disease (NAFLD) in humans and rodent model animals (Ding et al., 2004). This suggests that geese may have a unique mechanism that protects the liver from harmful effects associated with severe steatosis, which may have resulted from the long-term evolution of migratory ancestors. Usually, seasonal migratory birds store a large amount of fat in their liver and develop a physiological fatty liver before migration (Geng et al., 2015). The fat deposited in the liver is then gradually consumed during migration, which leads to a return of the fatty liver to a normal state. Recent studies have identified certain mechanisms responsible for the unique nature of goose liver. For example, the expression of complement genes and endoplasmic reticulum marker genes is generally suppressed in goose fatty liver compared to that in normal liver, whereas the expression of adiponectin receptor genes, mitochondria-related genes, and fatty acid desaturase genes is induced (Stanković et al., 2014). Revealing such unique protective mechanisms may provide new ideas for promoting the production of goose fatty liver and treating human fatty liver disease.

Gut microbes play an important role in the maintenance of normal physiological functions and disease occurrence in animals. First, intestinal bacteria possess enzymes that help degrade nutrients in the animal intestine and facilitate digestion and absorption (Wei et al., 2013). Second, intestinal bacteria can influence the intestinal structure, thus altering intestinal permeability; the chances of metabolites, toxins, and bacteria entering the blood; and immune homeostasis (Schwarzer et al., 2018). Furthermore, metabolites produced by intestinal bacteria (including degraded nutrients and harmful or bioactive substances synthesized by bacteria) can enter blood circulation through the intestine–liver axis and trigger systemic effects. The intestine–liver axis is the main pathway through which intestinal bacteria affect the liver function and pathology. Intestinal bacteria play a key role in the development of NAFLD in humans and rodents (Le Roy et al., 2013). During the development of NAFLD, intestinal bacteria can activate the TLR4 pathway and affect the expression of TNF-α; conversely, short-chain fatty acids produced by certain intestinal bacteria can inhibit the secretion of inflammatory cytokines, such as interleukin (IL) 2 (IL-2), IL-6, and tumor necrosis factor α (TNF-α), by suppressing the activation of the nuclear transcription factor κB (NF-κB) pathway. It is well-known that TNF-α and other inflammatory cytokines are essential for the development of non-alcoholic steatohepatitis (Park et al., 2007; Zhang et al., 2011).

Unlike in mammalian animals, in geese, the main source of fat is the liver. Although it is known that the composition of intestinal bacteria is affected by overfeeding, whether intestinal bacteria and their metabolites affect the formation of goose fatty liver and whether they provide protection against the deleterious effects of severe steatosis in geese remain unclear. A recent study that performed metagenomic sequencing analysis of intestinal bacteria on the 19th day of overfeeding showed that intestinal microbiota contributed to the regulation of lipid metabolism, amino acid metabolism, and immune disease pathways in goose fatty liver, preventing it from inflammation (Liu et al., 2016). In the present study, both 16S rRNA sequencing analysis and metagenomic sequencing analysis were employed to investigate the changes in bacterial composition in different parts of the intestinal tract (jejunum, ileum, and cecum) during the formation of goose fatty liver. *Lactobacillus* was supplemented in the diet to determine the relationship between the changes in gut microbiota and the formation of goose fatty liver. The findings of the present study may improve the overfeeding protocol and promote the production of goose fatty liver.

## Materials and Methods

### Experimental Animals and Sample Collection

This study included two animal experiments. In the first experiment, a total of 80 healthy Landes geese aged 70 days with similar average body weights from the same batch were divided into two groups and raised in Licheng Livestock and Poultry Breeding Co., Ltd. (Huai'an, Jiangsu, China). The control group, containing 48 geese, was fed *ad libitum* for 24 days, and the overfeeding group, containing 32 geese, was overfed for the same time. The overfeeding procedure has been previously described (Liu et al., 2016). The diet used was cooked maize, supplemented with 1% vegetable oil, 1% salt, and 0.2% vitamins. The geese were housed in cages under conventional conditions. At the beginning (the age of 70 days), 16 geese from the control group were weighed and sacrificed, and at the age of 82 and 94 days, 16 geese each from both the groups were weighed and sacrificed at each time point. The weights of the liver and abdominal fat were measured. The contents from the intestinal tract (jejunum, ileum, and cecum) were collected and transported on dry ice to Beijing Novogene Technology Co., Ltd. (Beijing, China) for 16S rRNA and metagenomic sequencing analyses. Based on 16S rRNA sequencing analysis, only the top-quality samples were subjected to metagenomic sequencing analysis, which included four content samples for each part of the intestinal tract per group at the age of 70 and 94 days.

In the second experiment (the experiment with *Lactobacillus* supplementation), 30 healthy Landes geese with similar body weight at 70 days of age were divided into two groups (*n* = 15): the overfeeding group and the overfeeding plus *Lactobacillus* supplementation. The geese in the overfeeding group were overfed the regular diet, as mentioned before, while those in the supplementation group were fed a regular diet supplemented with *Lactobacillus* (1‰ of the regular diet). The *Lactobacillus* was purchased from Taiwan Yaxin Biotechnology Co., Ltd. (the active *Lactobacillus* content ≥1.0 × 10^11^ per gram) and stored at 4°C. After 24 days of overfeeding, the experimental geese were individually weighed. Subsequently, the weights of the liver and abdominal fat were measured after the birds were sacrificed. Tissue samples were collected and stored in liquid nitrogen until further use. For 16S rRNA analysis, the cecum content of each bird was collected and transported on dry ice to Beijing Novogene Technology Co., Ltd. (*n* = 5). All animal protocols were approved by the Animal Welfare Committee of Yangzhou University (permission number SYXK (Su) IACUC 2016-0020).

### 16S rRNA Sequencing Analysis

Genomic DNA was extracted from the intestinal samples (*n* = 16) using the CTAB method, and the purity and concentration of the DNA samples were determined. Using the Phusion® High-Fidelity PCR Master Mix with GC Buffer (New England Biolabs) for PCR amplification, PCR was performed with the DNA samples and a pair of specific primers (forward: 5-AYTGGGYDTAAAGNG-3, reverse: 5-TACNVGGGTATCTAATCC-3). Subsequently, the DNA library for each sample was constructed with the PCR products using the TruSeq® DNA PCR-Free Sample Preparation Kit. The constructed libraries were quantified using Qubit and Q-PCR, followed by sequencing on the HiSeq2500 PE250 platform. The sequencing data were identified for each sample according to the barcode sequence, and barcode and primer sequences were removed. The reads from each sample were spliced together using FLASH, and the spliced sequences were filtered to obtain clean reads. All clean reads were clustered with 97% sequence identity into operational taxonomic units (OTUs) using Uparse software (Uparse v7.0.1001). Subsequently, species annotation was performed on the representative sequences of OTUs with a set threshold of 0.8–1 using the Mothur method with SILVA's SSUrRNA database. Metastats analysis was performed using the R software, and a permutation test between groups was performed to obtain *P*-values at each taxonomic level.

### Metagenomic Sequencing Analysis

The DNA samples extracted from intestinal contents were checked for purity and integrity using agarose gel electrophoresis (AGE) and quantified for concentration using Qubit (Shanghai, China). The DNA samples were then fragmented into ~350 bp fragments using a Covaris ultrasonic fragmentation apparatus (Shanghai, China). Subsequently, the DNA library for each sample was constructed through end repair, adapter addition, and PCR. The size of the fragments inserted into the constructed libraries was checked using Agilent 2100 Bioanalyzer (Agilent, Beijing, China), and the effective concentrations of the libraries were determined by Q-PCR. The effective library concentration should be >3 nM to ensure library quality. Sequencing of the libraries was performed using the Illumina PE150 sequencing platform. The quality of the raw data from the sequencing analysis was checked before filtering to obtain clean reads. The clean reads were used for sequence assembly and gene prediction using MetaGeneMark, followed by cataloging genes and obtaining gene abundance for each sample. By comparing with the MicroNR library, each gene (Unigene) was annotated for bacterial species. Subsequently, the bacterial abundances at different taxonomic levels were calculated using species information combined with gene abundance information, based on which the relative abundance profiles were plotted and analyzed using PCA. By comparing unigenes with functional databases (e.g., the KEGG database) using DIAMOND software (v0.7.9), the genes were functionally annotated, and the relative abundances of genes at different functional tiers and taxonomic levels were also calculated. Subsequently, Anosim between- and within-group variation analyses and Metastat and LEfSe analyses were performed.

### Statistical Analysis

Data are expressed as the mean ± standard error. Differences between groups were tested for significance using a *t*-test or one-way ANOVA in SPSS 16.0 (SPSS China, Shanghai, China) with *P* < 0.05 as a criterion of significance.

## Results

### Overfeeding Reduced the Biodiversity of Intestinal Microbiota

The average body weight of Landes geese increased from 3.72 kg at 70 days of age to 6.87 kg at 94 days of age after 24 days of overfeeding, while that of the control group increased from 3.71 to 4.42 kg. Correspondingly, the average liver weight and abdominal fat weight of the overfeeding group were 830.2 and 377.9 g, respectively, while those of the control group were 103.0 and 183.6 g. Compared with that of the control group, the ratio of liver weight to body weight of the overfeeding group significantly increased, indicating that overfeeding successfully induced the formation of goose fatty liver. Subsequently, 16S rRNA gene analysis was performed to determine the composition of the intestinal microbiota. The data showed that the number of OTUs in the control group ranged from 1,000 to 1,500, which was higher than that observed in the overfeeding group (all below 1,000 with the lowest in the cecum at 24 days of overfeeding) (Supplementary Figure 1), and the same regularity was found through the analysis of diversity (Supplementary Tables 1, 2). These results indicate that overfeeding reduced the biodiversity of the intestinal microbiota to some extent, particularly the cecal microbiota.

### Dominant Bacteria in Different Intestinal Tracts

**Figure 1:**
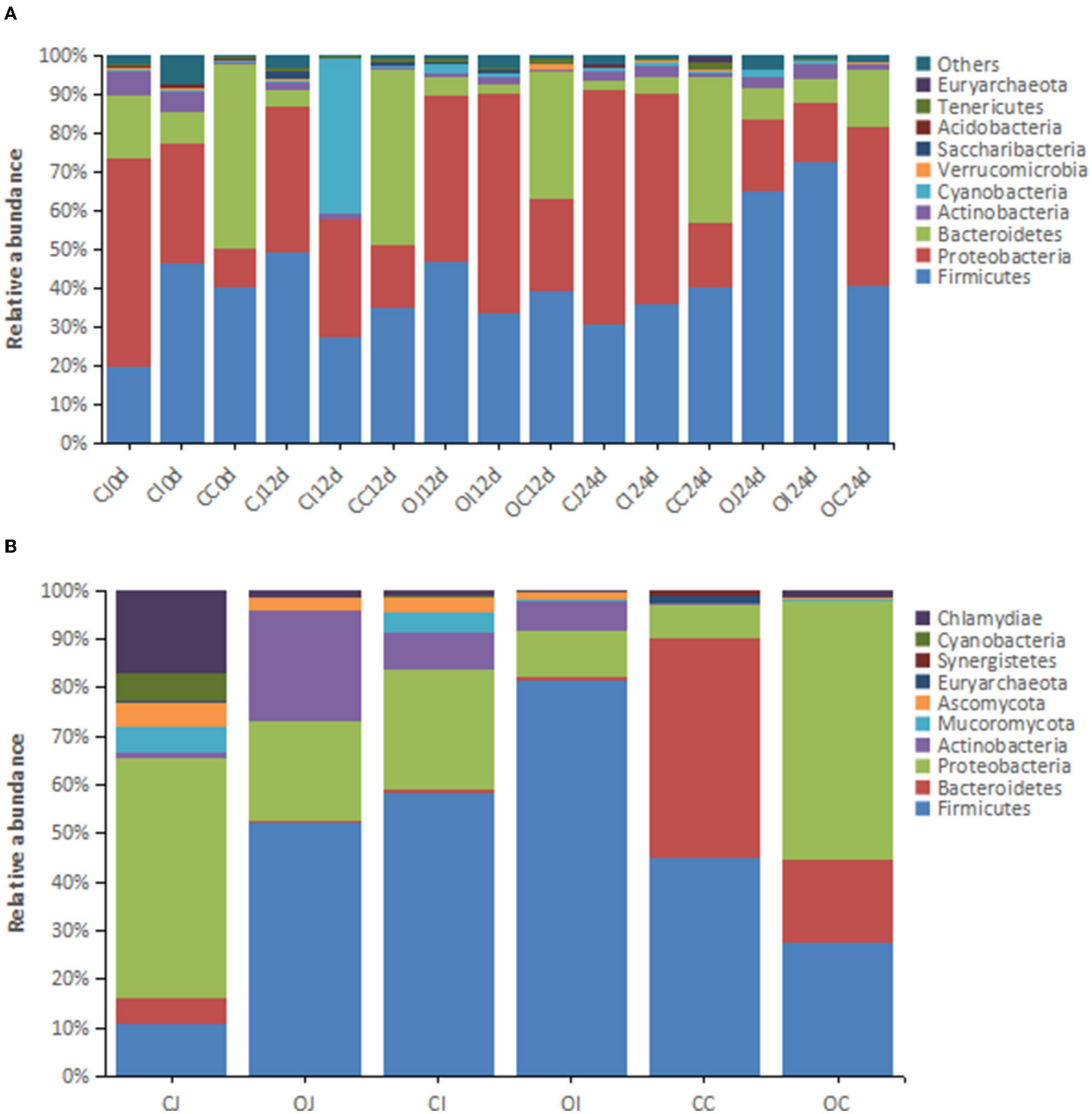
The histograms showing the relative abundance of the top 10 phyla in different intestinal tracts revealed by **(A)** 16S rRNA analysis (*n* = 16) and **(B)** metagenomic analysis (*n* = 4). Note: “CJ” and “OJ” denote the jejunal tracts of the control group and the overfeeding group, respectively. “CI” and “OI” denote the ileal tracts of the control group and the overfeeding group, respectively. “CC” and “OC” denote the cecal tracts of the control group and the overfeeding group, respectively.

A total of 55 phyla were identified by Mothur method-based clustering analysis of the 16S rRNA sequences of intestinal bacteria from the overfeeding and control groups of Landes geese at the age of 70, 82, and 94 days (Figure 1A). In terms of the average abundance of bacteria, the dominant phyla (the relative abundance > 1%) included *Firmicutes* (44.88%), *Proteobacteria* (32.96%), *Bacteroidetes* (15.06%), *Actinobacteria* (2.35%), and *Cyanobacteria* (1.12%), with the top three accounting for about 92% of the total bacterial population (Figure 1A). The dominant phyla showed varying relative abundance in different parts of the intestinal tract: *Proteobacteria* (30.90% in jejunum, 23.73% in ileum, and 10.24% in cecum) and *Bacteroidetes* (7.92% in jejunum, 6.03% in ileum, and 47.18% in cecum). The relative abundance of *Proteobacteria* in the cecum was markedly lower than that in the jejunum and ileum. In contrast, the relative abundance of *Bacteroidetes* was markedly higher. Metagenomic analysis showed that the dominant phyla (relative abundance > 1%) in the intestine were *Firmicutes* (46.00%), *Proteobacteria* (27.34%), *Bacteroidetes* (11.52%), *Actinobacteria* (6.36%), *Chlamydia* (3.49%), *Ascomycota* (2.04%), *Mucoromycota* (1.65%), and *Cyanobacteria* (1.10%) (Figure 1B). The result was similar to that of 16S rRNA analysis, which only differed for *Mucoromycota, Ascomycota*, and *Chlamydia*.

**Figure 2:**
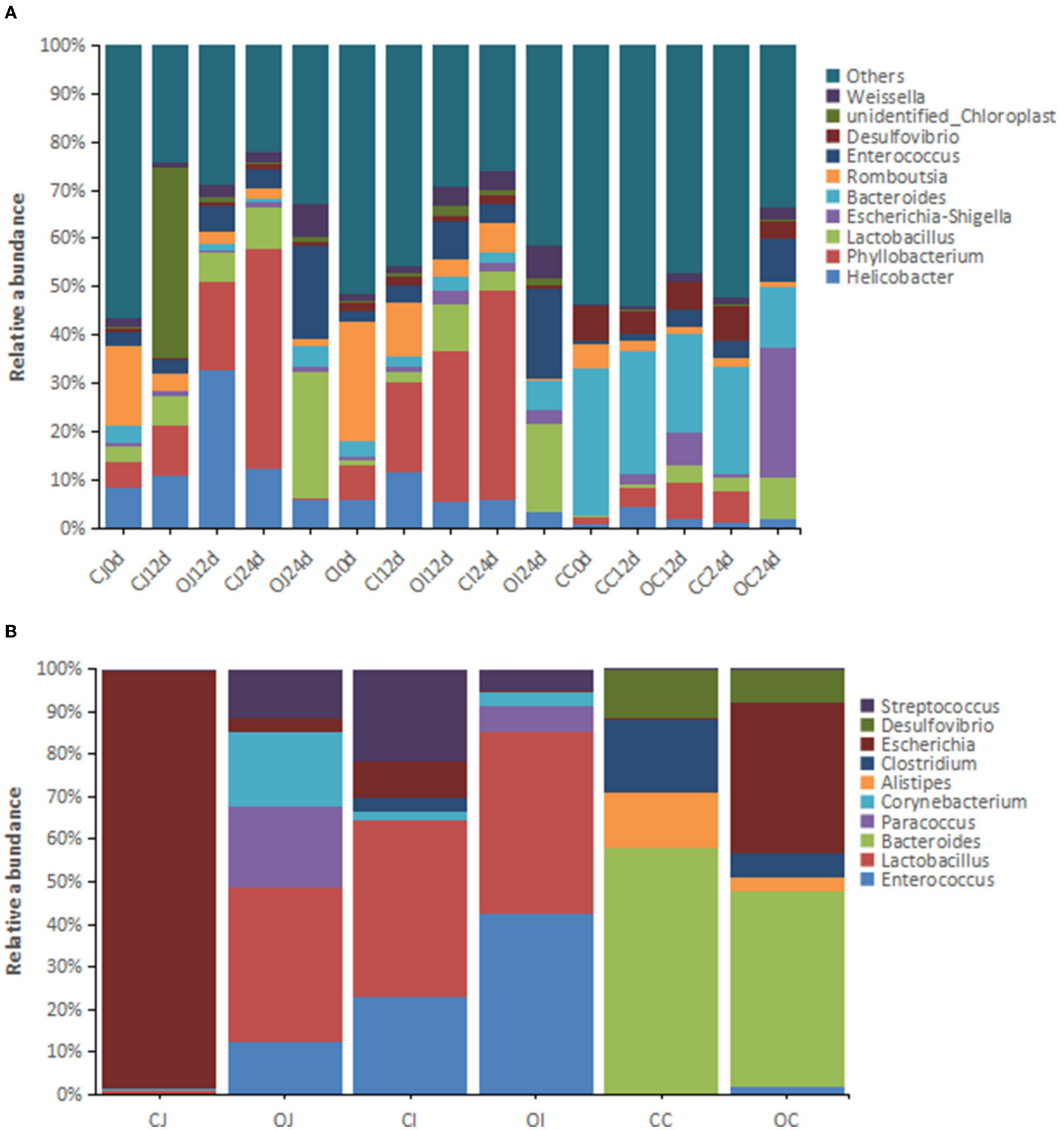
The histograms showing the relative abundance of the top 10 genera in different intestinal tracts revealed by **(A)** 16S rRNA analysis (*n* = 16) and **(B)** metagenomic analysis (*n* = 4). Note: “CJ” and “OJ” denote the jejunal tracts of the control group and the overfeeding group, respectively. “CI” and “OI” denote the ileal tracts of the control group and the overfeeding group, respectively. “CC” and “OC” denote the cecal tracts of the control group and the overfeeding group, respectively.

In terms of the dominant genera (relative abundance > 1%), 1,098 genera were detected by 16S rRNA analysis, which were predominantly anaerobic bacteria, with a small proportion of facultative anaerobic bacteria. Among them, *Phyllobacterium* (13.2%), *Bacteroides* (9.20%), *Helicobacter* (7.58%), *Lactobacillus* (6.72%), *Enterococcus* (5.88%), *Romboutsia* (5.55%), *Escherichia-Shigella* (2.61%), *Desulfovibrio* (2.61%), and *Weissella* (2.57%) were the most abundant intestinal bacteria, accounting for more than 50% of all bacteria (Figure 2A). The dominant genera with significant differences in relative abundance between different parts of the intestinal tract (jejunum, ileum, and cecum) included *Phyllobacterium, Helicobacter, Lactobacillus*, and *Romboutsia*. Metagenomic analysis showed that the dominant genera in the intestinal microbiota were *Lactobacillus* (6.03%), *Enterococcus* (4.67%), *Bacteroides* (4.21%), *Streptococcus* (1.31%), *Paracoccus* (1.27%), *Clostridium* (1.22%), *Escherichia (1.07%)*, and *Corynebacterium* (1.01%) (Figure 2B). Among them, *Enterococcus, Lactobacillus*, and *Bacteroides* were the dominant genera shared in both analyses.

### Relative Abundance of Dominant Bacteria Varied With Time

In terms of dominant phyla of bacteria, the relative abundance of the top three phyla (*Firmicutes, Proteobacteria*, and *Bacteroides*) in the control group did not show any obvious change over time in the cecum, whereas the relative abundance of *Firmicutes* gradually decreased, while the relative abundance of *Proteobacteria* gradually increased in the jejunum and ileum. The relative abundance of *Bacteroides* in the jejunum and ileum did not show any obvious change over time. These results suggest that the relative abundance of *Firmicutes* and *Proteobacteria* in the jejunum and ileum are liable to environmental factors, while the relative abundance of *Bacteroides* in the jejunum and ileum, as well as the relative abundance of the dominant phyla in the cecum, is tolerant to environmental changes and remain stable. On the other hand, in the overfeeding group, the relative abundance of the dominant phyla of bacteria in each part of the intestinal tract changed with time, i.e., the relative abundance of *Firmicutes* showed a decreasing trend at the earlier stage and an increasing trend at the later stage, while the relative abundance of *Proteobacteria* showed an increasing trend at the earlier stage and decreased at a later stage. The relative abundance of *Bacteroidetes* gradually decreased. The results show that *Firmicutes* and *Proteobacteria* in the jejunum and ileum of geese in the overfeeding state were more susceptible to environmental factors, while *Bacteroidetes* in the jejunum and ileum, as well as *Firmicutes* in the cecum, remained relatively stable.

In terms of dominant genera of bacteria, in the control group, the relative abundance of *Phyllobacterium, Lactobacillus*, and *Enterococcus* in each part of the intestinal tract increased with time, while that of *Romboutsia* decreased with time. In the overfeeding group, the relative abundance of *Lactobacillus, Enterococcus*, and *Weissella* in each part of the intestinal tract increased with time, whereas that of *Romboutsia* decreased.

### Effect of Overfeeding on the Relative Abundance of Dominant Bacteria

After overfeeding, the relative abundance of the phyla *Firmicutes* and *Actinobacteria* in the jejunum of Landes geese considerably increased, whereas that of *Proteobacteria* and *Bacteroidetes* decreased. In the ileum, the relative abundance of *Firmicutes* was obviously increased by overfeeding, whereas that of *Proteobacteria* decreased. In the cecum, the relative abundance of *Proteobacteria* increased considerably by overfeeding, whereas that of *Firmicutes* and *Bacteroidetes* decreased.

In terms of the dominant genus of bacteria, the relative abundance of *Enterococcus, Lactobacillus, Paracoccus*, and *Corynebacterium* in the jejunum of Landes geese considerably increased by overfeeding, while that of *Escherichia* decreased. In the ileum, the relative abundance of *Enterococcus, Lactobacillus, Paracoccus*, and *Corynebacterium* increased considerably by overfeeding. There was no decrease in the relative abundance of any of the genera in the ileum. In the cecum, the relative abundance of *Escherichia* considerably increased by overfeeding, whereas that of *Bacteroides, Clostridium*, and *Desulfovibrio* decreased.

### Significantly Different Relative Abundance of Intestinal Bacteria Between the Control and Overfeeding Groups

The relative abundance of several phyla was significantly different between the overfed and control geese, as identified by 16S rRNA analysis. At 12 days of overfeeding, the relative abundance of four phyla decreased significantly in the jejunum of the overfeeding group compared to that of the control group; however, none of the phyla displayed increased relative abundance (Supplementary Table 3). In the ileum of the overfeeding group, there were seven phyla with decreased relative abundance compared to that of the control group; in contrast, the relative abundance of *Fusobacteria* increased. In the cecum of the overfeeding group, the relative abundance of five phyla decreased compared with that of the control group; in contrast, the relative abundance of *Nitrospirae* increased. In the jejunum of the overfeeding group, there were five phyla with decreased relative abundance at 24 days of overfeeding compared to that of the control group. In contrast, *Firmicutes* and *Bacteroidetes* showed increased relative abundance. In the ileum of the overfeeding group, seven phyla showed decreased relative abundance compared to that of the control group; in contrast, the relative abundance of *Firmicutes* increased. In the cecum of the overfeeding group, eight phyla showed decreased relative abundance compared to that of the control group; in contrast, the relative abundance of *Proteobacteria* increased. The results suggest that prolongation of overfeeding time led to a decrease in the relative abundance of most of the differential phyla in the jejunum and cecum.

The major differential phyla of intestinal bacteria (relative abundance > 0.05%) identified by metagenomic analysis included *Actinobacteria* in the jejunum; *Firmicutes* in the ileum; and *Firmicutes, Bacteroidetes, Euryarchaeota, Synergistetes, Elusimicrobia, Chlamydiae, Ascomycota, Mucoromycota*, and *Chytridiomycota* in the cecum. *Firmicutes* in the ileum and *Bacteroidetes, Euryarchaeota, Synergistetes*, and *Elusimicrobia* in the cecum were consistently identified in both analyses; however, many of the differential phyla identified by 16S rRNA analysis were not identified by metagenomic analysis because of the very low abundance of other phyla and the technical differences between metagenomic analysis and 16S rRNA analysis (Supplementary Table 4).

The differential genera in each intestinal tract identified by 16S rRNA analysis between the control and overfeeding groups are listed in Supplementary Table 5. At 12 days of overfeeding, seven genera showed increased relative abundance in the jejunum of the overfeeding group compared to the control group; in contrast, 61 genera showed decreased relative abundance. In the ileum of the overfeeding group, 11 genera showed increased relative abundance compared to that of the control group, whereas 108 genera showed decreased relative abundance. In the cecum of the overfeeding group, 22 genera showed increased relative abundance compared to that of the control group, whereas 56 genera showed decreased relative abundance, and there were no dominant genera among them. At 24 days of overfeeding, 65 genera showed increased relative abundance in the jejunum of the overfeeding group compared to the control group, whereas 57 genera showed decreased relative abundance. In the ileum of the overfeeding group, 44 genera showed increased relative abundance in the overfeeding group compared to that of the control group, whereas 65 genera showed decreased relative abundance. In the cecum of the overfeeding group, 48 genera showed increased relative abundance compared to that of the control group, whereas 97 genera showed decreased relative abundance. Similar to the findings for the differential phyla, the number of differential genera in the jejunum and cecum increased with an increase in the overfeeding time.

The major differential genera (relative abundance > 0.05%) identified by metagenomic analysis are presented in Supplementary Table 6. *Lactobacillus* in the jejunum and *Bacteroides* in the cecum showed consistent results in both 16S rRNA and metagenomic analyses; however, many of the differential genera identified by 16S rRNA analysis were not consistent with those identified by metagenomic analysis because of the very low abundance of other genera and the technical differences between metagenomic analysis and 16S rRNA analysis.

### KEGG Pathway Analysis

Functional analysis of intestinal microbiota of Landes geese using the KEGG database showed that the highest number of Unigenes annotated in the category of “Cellular Processes” pathway was “Cellular Community” (9,463), that in the category of “Environmental Information Processing” pathway was “Membrane Transport” (20,511), that in the category of “Genetic Information Processing” pathway was “Transport” (20,037), that in the category of “Human Diseases” pathway was “Antimicrobial Resistance” (5,715), that in the category of “Metabolism” pathway was “Carbohydrate Metabolism” (38,644), and that in the category of “Organismal Systems” pathway was “Environmental Adaptation” (3,607) (Supplementary Figure 2). It is noteworthy that the major function of intestinal microbiota was related to metabolism, as the top three pathways with most Unigenes annotated in the category of “Metabolism” pathway were “Carbohydrate Metabolism” (38,644), “Global and Overview Maps” (36,066), and “Amino Acid Metabolism” (31,019).

**Figure 3:**
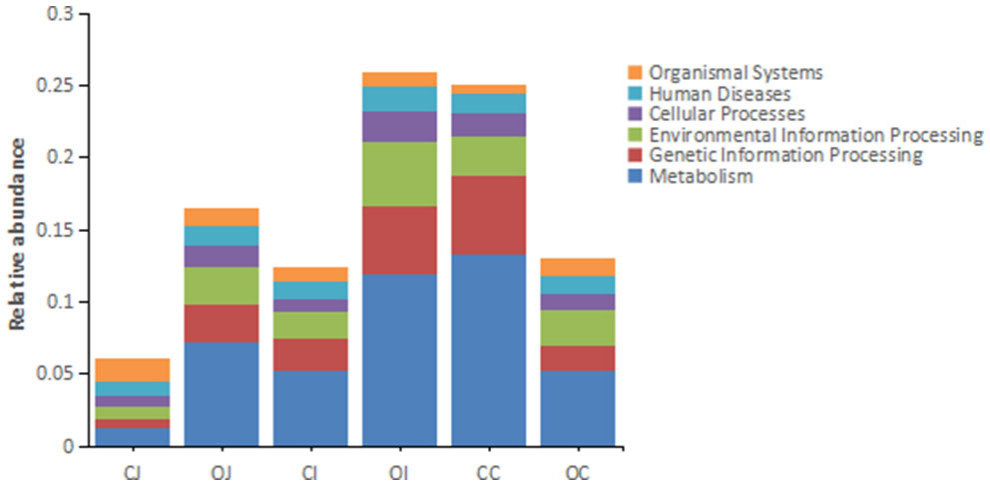
The histograms showing the relative abundance of each annotated function of different intestinal tracts at the first tier based on KEGG pathway analysis. Note: “CJ” and “OJ” denote the jejunal tracts of the control group and the overfeeding group, respectively. “CI” and “OI” denote the ileal tracts of the control group and the overfeeding group, respectively. “CC” and “OC” denote the cecal tracts of the control group and the overfeeding group, respectively.

Regarding the differential pathways of intestinal bacteria between the overfeeding group and the control group, there was an obvious effect of overfeeding on the metabolism of intestinal bacteria at the primary pathway level, i.e., the metabolism of bacteria in the jejunum and ileum was enhanced by overfeeding, while that in the cecum was inhibited by overfeeding (Figure 3). At the secondary pathway level, there was no differential pathway in the jejunum; in contrast, there were three differential pathways in the ileum, including “endocrine system” in “organismal systems,” “signal transduction” in “environmental information processing,” and “cellular community” in “cellular processes,” and 37 differential pathways in the cecum, mainly including “global and overview maps,” “carbohydrate metabolism,” “amino acid metabolism,” and “energy metabolism” in “metabolism” among other (Supplementary Table 7). At the tertiary pathway level, there was only one in the jejunum, i.e., “African trypanosomiasis,” and six in the ileum, including “phosphatidylinositol signaling system,” “thyroid hormone synthesis,” “cutin, suberin, and wax biosynthesis,” “RNA transport,” “PPAR signaling pathway,” “NOD-like receptor signaling pathway,” and 306 in the cecum, mainly including “biosynthesis of amino acids,” “carbon metabolism,” “purine metabolism,” “ribosome,” “pyrimidine metabolism,” “amino sugar and nucleotide sugar metabolism,” “glycolysis,” “pyruvate metabolism,” “oxidative phosphorylation,” “olfactory transduction,” etc. (Supplementary Table 8).

### Effects of Supplemented *Lactobacillus* on the Overfed Geese

**Figure 4:**
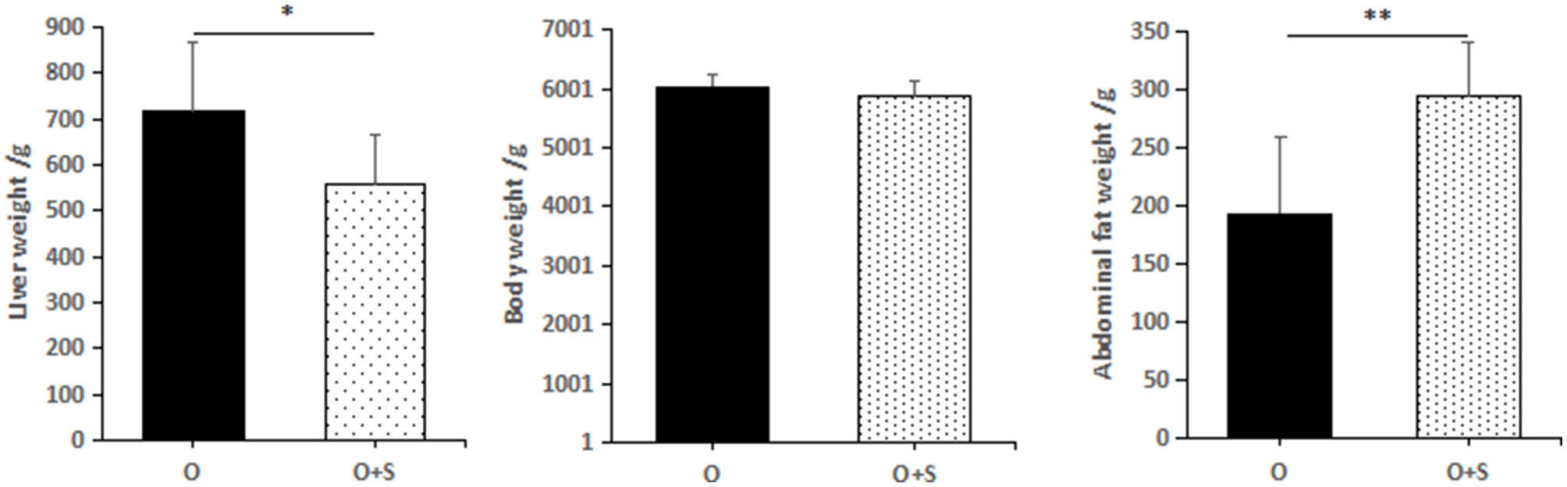
The effects of lactobacillus supplementation on body weight, liver weight, and abdominal fat weight of Landes geese. Note: the geese were overfed for 24 days. “O” and “O + S” denote the overfeeding group and the overfeeding + supplementing group, respectively.

Comparing the overfeeding group with the supplementation group (the overfed geese supplemented with *Lactobacillus*), it was found that supplementation with *Lactobacillus* significantly increased abdominal weight (190 ± 70 g in the overfeeding group and 290 ± 60 g in the supplementation group), but significantly decreased liver weight (715 ± 151 g in the overfeeding group and 556 ± 111 g in the supplementation group). There was no significant difference in the body weight (6,030 ± 220 g in the overfeeding group and 5,870 ± 256 g in the supplementation group) between the groups (Figure 4).

**Figure 5:**
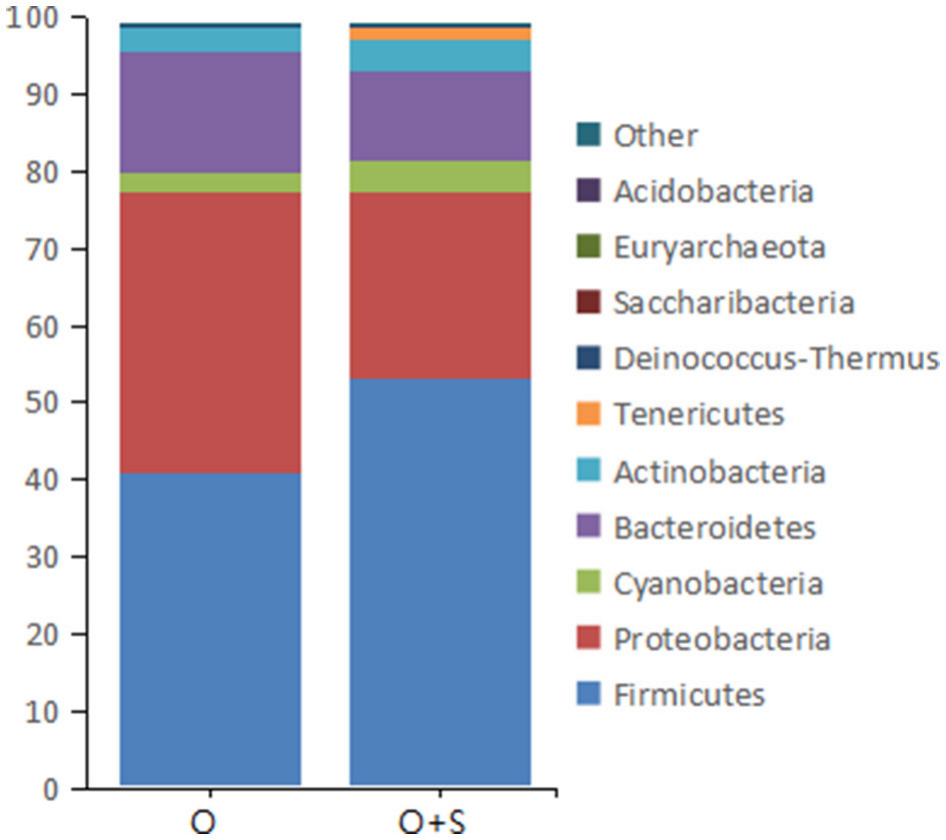
The histograms showing the relative abundance of the top 10 phyla in the cecum of the overfeeding group and the supplementing group of geese (*n* = 15). Note: the relative abundance was acquired from 16S rRNA analysis. The supplementing group of geese was overfed with the diet supplemented with lactobacillus. “O” and “O + S” denote the overfeeding group and the overfeeding + supplementing group, respectively.

With regard to the effects of supplementation of *Lactobacillus* on bacterial phyla in the cecum, the data showed that the relative abundance of *Proteobacteria* and *Bacteroidetes* decreased in the supplementation group compared to the overfeeding group; in contrast, the relative abundance of *Firmicutes, Cyanobacteria*, and *Actinobacteria* was increased (Figure 5).

**Figure 6:**
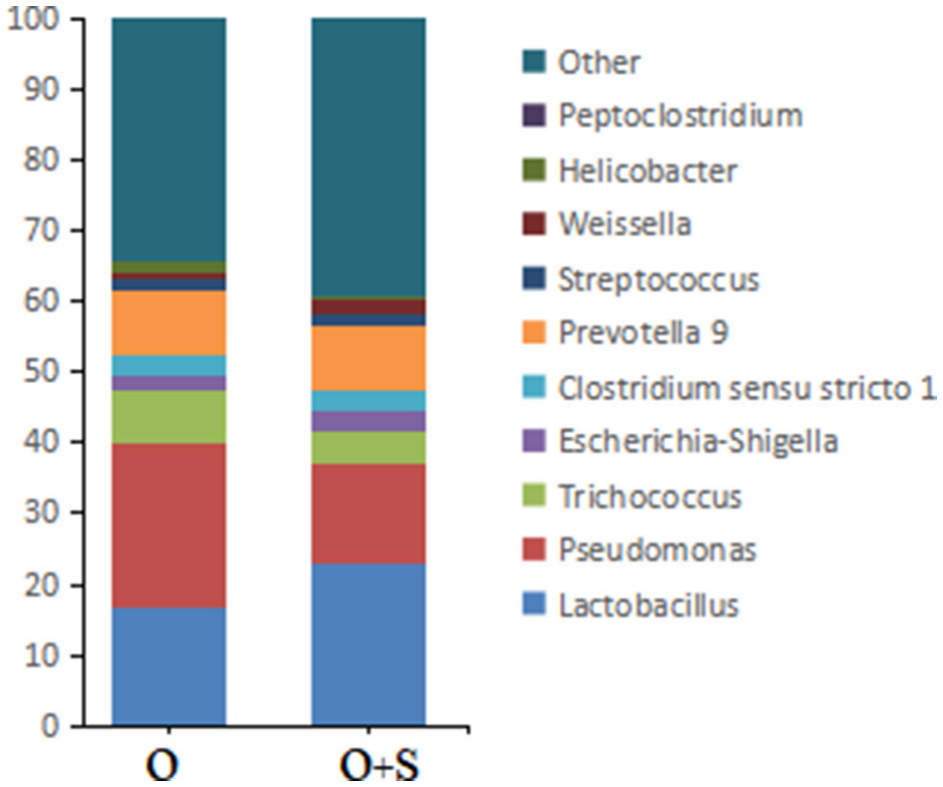
The histograms showing the relative abundance of TOP 10 genera in the cecum of the overfeeding group and the supplementing group of geese (n = 15). Note: the relative abundance was acquired from 16S rRNA analysis. “O” and “O+S” denote the overfeeding group and the overfeeding + supplementing group, respectively.

With regard to the effects of supplementation of *Lactobacillus* on bacterial genera in the cecum, the data showed that the relative abundance of *Pseudomonas, Trichococcus, Helicobacter*, and *Peptoclostridium* decreased in the supplementation group compared to the overfeeding group; in contrast, the relative abundance of *Lactobacillus, Peptoclostridium, Clostridium sensu stricto 1, Escherichia-Shigella, Weissella*, and *Streptococcus* increased (Figure 6).

## Discussion

The large amount of carbohydrates ingested by Landes geese through 3–4 weeks of overfeeding can lead to the formation of goose fatty liver, which is usually 8–10 times heavier than the normal liver. There is evidence to support that the formation of goose fatty liver is associated with changes in the intestinal microbiota (Liu et al., 2016), but little is known about the changes in the composition and function of the intestinal microbiota during the formation of goose fatty liver and the effect of probiotic supplementation on the formation of goose fatty liver (Hermier et al., 1994). In this study, 16S rRNA and metagenomic sequencing analyses of intestinal microbiota and the supplementation of *Lactobacillus* were performed.

This study revealed that overfeeding reduced the biodiversity of the intestinal microbiota, especially in the cecum, which is consistent with a previous report (Liu et al., 2016). Previous studies have also reported that the formation of fatty liver in humans and other animals is accompanied by reduced biodiversity of the intestinal microbiota (Nowland et al., 2019). In this study, the dominant phyla of intestinal bacteria in Landes geese were *Firmicutes, Proteobacteria, Bacteroidetes*, and *Actinobacteria*. This is in line with previous reports showing that the dominant phylum of intestinal bacteria accounts for more than 90% of the total intestinal microbes in other goose breeds, such as the Yangzhou goose (Lei et al., 2019), Sichuan white goose (Cheng et al., 2020), and Yan goose (Manman et al., 2019). The relative abundance of the dominant phylum varied with different intestinal tracts, *i.e*., the relative abundance of *Ascomycetes* was low in the jejunum and ileum but high in the cecum, whereas the relative abundance of *Bacteroidetes* was the opposite. This difference may be related to the physiological environment in different parts of the intestinal tract, such as pH, degree of food digestion, and nutritional composition, which may affect the growth and proliferation of dominant bacteria to different degrees (Zhu et al., 2015). Second, the relative abundance of the dominant phylum of intestinal bacteria in Landes geese also changed during the entire duration of the overfeeding. In the absence of overfeeding, the change in the relative abundance of the dominant phylum over time may be mainly due to the change in feed composition (the experimental diet consisted of maize, which was quite different from the diet used before the experiment). In addition, the effects of physiological and environmental changes in the intestine on the proliferation of intestinal microbes should not be excluded, as the geese underwent a large change in body weight and organ development during the 24-day experimental period. Third, the relative abundance of *Firmicutes* in the jejunum and ileum over time under the states of overfeeding or no overfeeding showed a trend opposite to that of Proteobacteria. More data are required to demonstrate whether this was due to an antagonistic relationship between the two or due to their different responses to external environmental changes.

The study also revealed the dominant genera of intestinal bacteria in Landes geese, which included *Phyllobacterium, Bacteroides, Helicobacter, Lactobacillus, Enterococcus*, and *Romboutsia*. Most of the dominant genera of bacteria (e.g., *Bacteroides, Lactobacillus*, and *Enterococcus*) are probiotics, which remain relatively abundant for the maintenance of intestinal health (Nazila et al., 2014; Zitnan et al., 2019; Vernay et al., 2020). The relative abundance of dominant genera is not only related to their location in the intestinal tract and the duration of feeding, but also to the effect of overfeeding. However, the species composition of the dominant genera remained relatively constant, suggesting that the dominant genera of intestinal bacteria play an important role in maintaining intestinal health and function in geese. Furthermore, the relative abundance of the dominant genera increased with the increase in feeding or overfeeding time, especially in the jejunum and ileum. Whether the increase in the relative abundance of dominant bacterial genera caused by overfeeding is beneficial for goose fatty livers to avoid inflammatory responses caused by harmful bacteria needs to be verified in the future. However, unlike in the jejunum and ileum, overfeeding appeared to cause an increase in the relative abundance of harmful bacterial genera (e.g., *Escherichia-Shigella* and *Veillonella*) and a decrease in the relative abundance of beneficial bacterial genera (e.g., *Bacteroides, Lactobacillus*, and *Enterococcus*) in the cecum (Pustelny et al., 2015; Liu et al., 2017). A possible explanation for this is that the migration of harmful genera from the jejunum and ileum to the cecum may facilitate the excretion of harmful bacteria. In addition to causing a change in the relative abundance of the dominant phylum or genera of bacteria, overfeeding also significantly altered the relative abundance of other intestinal bacteria, leading to an overall increase in the relative abundance of beneficial bacteria and a decrease in the relative abundance of harmful bacteria. Such changes would reduce the incidence of the inflammatory response in the goose fatty liver by improving the intestinal structure and immune barrier and inhibiting the production and permeation of harmful substances. Indeed, it has been reported that overfeeding does not increase LPS levels in the blood of geese (Ying et al., 2020).

The dominant bacteria in the goose intestine identified in this study have different biological roles. From the dominant phylum of bacteria, *Firmicutes* are mainly *Clostridia* and play an important role in the protein degradation of nutrients, digestion and absorption of starch, and synthesis of short-chain fatty acids (Shaona et al., 2020). *Firmicutes* produce short-chain fatty acids, which are important energy sources for host animals and contribute to the maintenance of intestinal microecological stability and regulation of animal physiological functions (Cassir et al., 2016). Fatty acids not only promote the growth and proliferation of beneficial bacteria but also inhibit the adverse effects of harmful bacteria (Zhu et al., 2015). Fatty liver formation in geese has been shown to be mainly due to the disruption of the balance between fat synthesis, fat transport, and β-oxidation of fatty acids in the liver, which causes abnormal fat deposition in the liver (Fournier et al., 1997). An increase in *Firmicutes* usually indicates an increase in the number of beneficial bacteria in the intestine (La Rosa et al., 2019). *Bacteroidetes* have a strong ability to break down fibrous materials and participate in sugar metabolism by encoding polysaccharidases. Overfed geese need to consume and digest a large amount of feed in a short period of time, and the increase in the relative abundance of *Bacteroidetes* indicates that microbiota can adapt to this dietary change by altering the composition of intestinal microbes, thus this increase is a positive adaptation. Changes in the relative abundance of *Proteobacteria* are indicative of dysbiosis in the intestinal microbiota. An increase in the relative abundance of *Proteobacteria* is often accompanied by dysregulation of intestinal microecology in the presence of metabolic disorders (Shin et al., 2015). The decrease in the relative abundance of *Proteobacteria* also is a positive adaptation in the overfed geese. The abundance of *Actinobacteria*, an important phylum in the intestine, has a significant influence on the health of host animals. A decrease in the relative abundance of *Actinobacteria* is often observed in patients with cirrhosis, particularly in the genus *Bifidobacterium* in the *Actinobacteria* phylum (Zhou et al., 2017). Using *Bifidobacterium* in the treatment of NAFLD can significantly improve the liver function of patients (Zhang, 2017). Among the dominant genera of bacteria, *Lactobacillus* is an important beneficial genus in the intestinal tract. Some studies have shown that *Lactobacillus* can regulate intestinal barrier function, defensin production, and inflammatory responses, and inhibit the invasion of harmful bacteria (Artis, 2008). Phyllobacterium is a heterotrophic genus of bacteria that uses various sugars or organic acid salts as carbon sources (Larcher et al., 2008). As an important genus for plant digestion, Phyllobacterium can break down large amounts of carbohydrates during overfeeding.

In this study, KEGG pathway analysis of the intestinal bacteria showed that the functions of the intestinal bacteria were mainly in nutrient metabolism, processing of environmental information, and resistance to antibiotics. Similar to symbiotic bacteria, intestinal bacteria play a role in nutrient metabolism, both to meet the needs of their own growth and proliferation and to facilitate the digestion and absorption of nutrients by the host. In addition to the basic function of participating in nutrient metabolism, intestinal bacteria need to have the ability to process external environmental signals to better adapt to the changing external environment. In addition, intestinal bacteria may face inter-bacterial resistance and antibiotic effects from the feed source; therefore, intestinal bacteria must also have the ability to resist antibiotics.

Interestingly, the changes in the composition, structure, and relative abundance of intestinal bacteria caused by overfeeding were also reflected in the corresponding functional changes. First, overfeeding affects the metabolic function of intestinal bacteria. Overfeeding can enhance the metabolism of intestinal bacteria in the jejunum and ileum but weaken the metabolism of intestinal bacteria in the cecum. This also indicates that the role of intestinal bacteria in nutrient metabolism is stronger in the jejunum and ileum than in the cecum, which is consistent with the physiological functions of each intestinal tract. In addition, our unpublished data suggest that overfeeding leads to a significant reduction in goose cecal weight, which further supports the idea that the cecum and its intestinal bacteria play a diminished role in nutrient metabolism. Second, the environmental information processing function of intestinal bacteria is also affected by overfeeding, and this effect may be related to the fact that overfeeding can change the intestinal environment, as well as the composition and abundance of intestinal bacteria. Third, it is worth mentioning that overfeeding can affect the hormone synthesis of intestinal bacteria. Previous studies have shown that the metabolites of certain intestinal bacteria may be substrates for hormone synthesis in the host. Whether these intestinal bacteria are involved in the formation of goose fatty liver by influencing hormone synthesis in the host should be verified in the future.

This study and previous studies suggest that the relative abundance of *Lactobacillus* in the intestine of overfed geese is increased significantly, and that lactic acid, a metabolite of *Lactobacillus*, can inhibit the complementary expression in goose fatty liver, thus helping goose fatty liver to avoid inflammatory responses (Huang et al., 2004; Wang et al., 2007). However, the effect of supplemented *Lactobacillus* on other indexes, such as the weight of goose fatty liver, is unclear. This study indicates that supplementation with *Lactobacillus* can reduce body weight and fatty liver weight but increase the weight of abdominal fat in geese, which implies that supplemented *Lactobacillus* might affect the transport of nascent fat from the liver to abdominal fat. Whether this effect is related to lactic acid, a metabolite of *Lactobacillus*, or to changes in the abundance of other intestinal bacteria following *Lactobacillus* supplementation should be investigated in future studies.

## Conclusion

In conclusion, the dominant bacterial species in the intestinal tract of geese were relatively stable, but their relative abundances were influenced by their location in different intestinal tracts, overfeeding, and overfeeding time. This influence changes the metabolic flux of intestinal bacteria, especially in nutrient metabolism, environmental information processing, and antibiotic resistance. The reduction in the biodiversity of intestinal bacteria and changes in the abundance of bacteria caused by overfeeding may be closely related to the formation of fatty liver in geese.

## Data Availability Statement

The 16S rRNA sequencing data can be found in online repositories. The names of the repository/repositories and accession number(s) can be found below: NCBI - PRJNA820543. The metagenomic sequencing data can be found in the Genome Sequence Archive of the National Genomics Data Center (https://bigd.big.ac.cn/gsa/browse/CRA007156) with accession number CRA007156.

## Ethics Statement

This study was approved by the Institutional Animal Care and Use Committee (IACUC) of Yangzhou University.

## Author Contributions

TG and DG conceived this study. KW, LL, and MZ designed the study, performed the experiment, collected samples, and carried out data analysis. KW drafted the manuscript. TG revised the manuscript. DG finalized the manuscript. All authors read and approved the final version of this manuscript.

## Funding

This work was supported by the National Nature Science Foundation of China (31672405, 32172756, 31972546, and 32172717) (Beijing, China) and the JBGS Project of Seed Industry Revitalization in Jiangsu Province (JBGS[2021]030 and JBGS[2021]105) (Jiangsu, China).

## Conflict of Interest

The authors declare that the research was conducted in the absence of any commercial or financial relationships that could be construed as a potential conflict of interest.

## Publisher's Note

All claims expressed in this article are solely those of the authors and do not necessarily represent those of their affiliated organizations, or those of the publisher, the editors and the reviewers. Any product that may be evaluated in this article, or claim that may be made by its manufacturer, is not guaranteed or endorsed by the publisher.
