# Supplementary material for: The Changes in Microbiotic Composition of Different Intestinal Tracts and the Effects of Supplemented Lactobacillus During the Formation of Goose Fatty Liver
Source: Front Microbiol. 2022 Jul 18;13:906895. doi: 10.3389/fmicb.2022.906895 (PMC9339986; doi:10.3389/fmicb.2022.906895)
Supplement: Supplementary file 1 [file Table_1.docx]

**Supplementary Table 1.** Alpha Indices Statistics

|  | **Observed species** | **shannon** | **simpson** | **chao1** | **ACE** | **PD whole_tree** |
| --- | --- | --- | --- | --- | --- | --- |
| CJ0d | 1,214.06 | 5.70 | 0.90 | 1,537.48 | 1,589.69 | 94.02 |
| CJ12d | 831.88 | 4.77 | 0.86 | 1,091.22 | 1,128.51 | 69.40 |
| CJ24d | 745.56 | 3.63 | 0.70 | 998.97 | 1,030.59 | 72.69 |
| OJ12d | 694.13 | 4.18 | 0.78 | 881.57 | 931.28 | 67.39 |
| OJ24d | 660.25 | 4.74 | 0.88 | 866.11 | 899.00 | 50.47 |
| CI0d | 1,028.94 | 5.52 | 0.88 | 1,273.26 | 1,312.79 | 72.71 |
| CI12d | 1,077.00 | 5.62 | 0.90 | 1,312.16 | 1,349.83 | 78.94 |
| CI24d | 782.94 | 4.14 | 0.75 | 1,037.36 | 1,072.93 | 58.85 |
| OI12d | 686.13 | 4.46 | 0.82 | 884.81 | 920.62 | 54.86 |
| OI24d | 692.50 | 5.00 | 0.89 | 908.57 | 938.46 | 58.16 |
| CC0d | 909.19 | 6.57 | 0.96 | 1,091.31 | 1,097.55 | 58.22 |
| CC12d | 908.06 | 6.60 | 0.96 | 1,106.96 | 1,128.56 | 61.31 |
| CC24d | 837.19 | 6.46 | 0.96 | 1,017.22 | 1,042.72 | 57.75 |
| OC12d | 742.00 | 5.97 | 0.94 | 950.82 | 968.05 | 53.32 |
| OC24d | 462.69 | 4.24 | 0.83 | 609.48 | 632.79 | 41.39 |

Note: ‘CJ’ and ‘OJ’ denote the jejunal tracts of the control group and the overfeeding group, respectively (n=16). ‘CI’ and ‘OI’ denote the ileal tracts of the control group and the overfeeding group, respectively. ‘CC’ and ‘OC’ denote the cecal tracts of the control group and the overfeeding group, respectively.
