# Supplementary material for: The Changes in Microbiotic Composition of Different Intestinal Tracts and the Effects of Supplemented Lactobacillus During the Formation of Goose Fatty Liver
Source: Front Microbiol. 2022 Jul 18;13:906895. doi: 10.3389/fmicb.2022.906895 (PMC9339986; doi:10.3389/fmicb.2022.906895)
Supplement: Supplementary file 2 [file Table_2.docx]

**Supplementary Table 2.** Adonis analysis of differences between groups

| **Vs_group** | **Df** | **SumsOfSqs** | **MeanSqs** | **F.Model** | **R2** | **Pr(>F)** |
| --- | --- | --- | --- | --- | --- | --- |
| CI0d-CJ0d | 1(30) | 0.54(6.61) | 0.54(0.22) | 2.47 | 0.076(0.92) | 0.029 |
| CI0d-CC0d | 1(30) | 2.46(3.33) | 2.46(0.11) | 22.14 | 0.42(0.57) | 0.001 |
| CI0d-CI24d | 1(30) | 1.82(4.31) | 1.82(0.14) | 12.68 | 0.29(0.70) | 0.001 |
| CI0d-OI12d | 1(30) | 1.93(4.64) | 1.93(0.15) | 12.47 | 0.29(0.70) | 0.001 |
| CI0d-OI24d | 1(30) | 2.83(5.69) | 2.83(0.18) | 14.94 | 0.33(0.66) | 0.001 |
| CJ0d-CC0d | 1(30) | 3.59(4.55) | 3.59(0.15) | 23.64 | 0.44(0.56) | 0.001 |
| CJ0d-OJ24d | 1(30) | 2.49(5.99) | 2.49(0.19) | 12.46 | 0.29(0.70) | 0.001 |
| CI12d-CI0d | 1(29) | 0.67(4.20) | 0.67(0.14) | 4.62 | 0.13(0.86) | 0.001 |
| CI12d-CC12d | 1(29) | 2.06(2.51) | 2.06(0.086) | 23.76 | 0.45(0.55) | 0.001 |
| CI12d-CI24d | 1(29) | 0.87(3.13) | 0.87(0.10) | 8.08 | 0.22(0.78) | 0.002 |
| CI12d-OI12d | 1(29) | 0.94(3.46) | 0.94(0.11) | 7.88 | 0.21(0.78) | 0.001 |
| CI12d-OC12d | 1(29) | 1.73(3.68) | 1.73(0.13) | 13.67 | 0.32(0.68) | 0.001 |
| CI12d-OI24d | 1(29) | 2.91(4.51) | 2.91(0.15) | 18.71 | 0.39(0.60) | 0.001 |
| CC12d-CC0d | 1(30) | 0.49(1.64) | 0.49(0.054) | 9.02 | 0.23(0.76) | 0.001 |
| CC12d-CC24d | 1(30) | 0.46(2.35) | 0.46(0.078) | 5.92 | 0.16(0.83) | 0.001 |
| CI24d-OI24d | 1(30) | 2.89(4.61) | 2.89(0.15) | 18.83 | 0.38(0.61) | 0.001 |
| CJ24d-CJ0d | 1(31) | 2.08(5.85) | 2.08(0.18) | 11.04 | 0.26(0.73) | 0.001 |
| CJ24d-CI24d | 1(31) | 0.19(3.56) | 0.19(0.11) | 1.67 | 0.051(0.94) | 0.081 |
| CJ24d-CC24d | 1(31) | 3.29(3.29) | 3.29(0.10) | 30.95 | 0.49(0.50) | 0.001 |
| CJ24d-OJ24d | 1(31) | 3.15(4.02) | 3.15(0.13 | 24.31 | 0.43(0.56) | 0.001 |
| CC24d-CC0d | 1(30) | 1.05(1.99) | 1.05(0.067) | 15.83 | 0.34(0.65) | 0.001 |
| OI12d-CI24d | 1(30) | 0.39(3.57) | 0.39(0.12) | 3.34 | 0.10(0.89) | 0.002 |
| OI12d-OI24d | 1(30) | 1.77(4.94) | 1.77(0.16) | 10.73 | 0.26(0.73) | 0.001 |
| OJ12d-CJ0d | 1(30) | 1.56(6.11) | 1.56(0.20) | 7.66 | 0.20(0.79) | 0.001 |
| OJ12d-CJ24d | 1(31) | 0.99(4.13) | 0.99(0.13) | 7.44 | 0.19(0.81) | 0.001 |
| OJ12d-OI12d | 1(30) | 0.83(4.14) | 0.83(0.14) | 6.03 | 0.16(0.83) | 0.001 |
| OJ12d-OC12d | 1(30) | 2.43(4.36) | 2.43(0.15) | 16.77 | 0.36(0.64) | 0.001 |
| OJ12d-OJ24d | 1(30) | 2.16(4.27) | 2.16(0.14) | 15.18 | 0.34(0.66) | 0.001 |
| OC12d-CC0d | 1(30) | 1.58(2.81) | 1.58(0.093) | 16.91 | 0.36(0.64) | 0.001 |
| OC12d-CC12d | 1(30) | 0.77(3.16) | 0.77(0.11) | 7.36 | 0.19(0.80) | 0.001 |
| OC12d-CC24d | 1(30) | 0.47(3.52) | 0.47(0.12) | 4.01 | 0.11(0.88) | 0.002 |
| OC12d-OI12d | 1(30) | 1.64(4.11) | 1.65(0.14) | 12.01 | 0.28(0.71) | 0.001 |
| OI24d-OJ24d | 1(30) | 0.24(5.07) | 0.24(0.17) | 1.42 | 0.045(0.95) | 0.134 |
| OC24d-CC0d | 1(30) | 4.37(3.34) | 4.37(0.11) | 39.27 | 0.56(0.43) | 0.001 |
| OC24d-CC12d | 1(30) | 3.65(3.70) | 3.65(0.12) | 29.61 | 0.49(0.50) | 0.001 |
| OC24d-CC24d | 1(30) | 3.33(4.05) | 3.33(0.13) | 24.66 | 0.45(0.54) | 0.001 |
| OC24d-OC12d | 1(30) | 1.89(4.86) | 1.89(0.16) | 11.65 | 0.28(0.72) | 0.001 |
| OC24d-OI24d | 1(30) | 1.05(5.69) | 1.05(0.18) | 5.56 | 0.16(0.84) | 0.001 |
| OC24d-OJ24d | 1(30) | 1.68(4.78) | 1.68(0.15) | 10.55 | 0.26(0.74) | 0.001 |

Note: ‘CJ’ and ‘OJ’ denote the jejunal tracts of the control group and the overfeeding group, respectively (n=16). ‘CI’ and ‘OI’ denote the ileal tracts of the control group and the overfeeding group, respectively. ‘CC’ and ‘OC’ denote the cecal tracts of the control group and the overfeeding group, respectively. Df=Degree of freedom; Sums Of Sqs=Sum of Squares of Deviations; MeanSqs=Sums Of Sqs / Df; F.Model= F test value; R2=Sums Of Sqs / group variance; Pr=*P*-value.
