# Supplementary material for: The Changes in Microbiotic Composition of Different Intestinal Tracts and the Effects of Supplemented Lactobacillus During the Formation of Goose Fatty Liver
Source: Front Microbiol. 2022 Jul 18;13:906895. doi: 10.3389/fmicb.2022.906895 (PMC9339986; doi:10.3389/fmicb.2022.906895)
Supplement: Supplementary file 3 [file Table_3.docx]

**Supplementary Table 3.** The relative abundance of the main differential bacteria (≥1%) at the phylus level between the control group and the overfeeding group

|  | **Phylum** | **Control (%)** | **Overfeeding (%)** | ***P*-value** |
| --- | --- | --- | --- | --- |
| 12d Jejunum | *Cyanobacteria* | 7.21±3.07 | 0.887±0.368 | 4.30×10^-2^ |
| 12d Cecum | *Bacteroidetes* | 44.8±2.95 | 32.7±3.14 | 6.70×10^-3^ |
| 24d Jejunum | *Proteobacteria* | 61.9±4.85 | 14.9±3.30 | 3.19×10^-9^ |
|  | *Firmicutes* | 29.4±4.68 | 72.7±3.61 | 1.99×10^-8^ |
|  | *Nitrospirae* | 2.41±0.663 | 0.855±0.313 | 3.66×10^-2^ |
|  | *Bacteroidetes* | 2.35±0.652 | 6.12±1.68 | 3.90×10^-2^ |
| 24d Ileum | *Proteobacteria* | 54.2±4.72 | 18.7±3.56 | 8.43×10^-7^ |
|  | *Firmicutes* | 35.9±3.40 | 64.7±4.48 | 1.00×10^-5^ |
| 24d Cecum | *Bacteroidetes* | 37.7±2.01 | 14.5±4.23 | 1.68×10^-5^ |
|  | *Proteobacteria* | 16.4±1.28 | 40.9±4.51 | 7.46×10^-6^ |
|  | *Tenericutes* | 1.73±0.165 | 0.0682±0.0467 | 4.38×10^-11^ |
|  | *Euryarchaeota* | 1.17±0.176 | 2.00×10^-3^±1.47×10^-3^ | 1.38×10^-7^ |

Note: the relative abundance of intestinal bacteria was determined by 16S rRNA analysis. n=16.
