# Supplementary material for: The Changes in Microbiotic Composition of Different Intestinal Tracts and the Effects of Supplemented Lactobacillus During the Formation of Goose Fatty Liver
Source: Front Microbiol. 2022 Jul 18;13:906895. doi: 10.3389/fmicb.2022.906895 (PMC9339986; doi:10.3389/fmicb.2022.906895)
Supplement: Supplementary file 4 [file Table_4.docx]

**Supplementary Table 4.** The relative abundance of differential bacteria in different intestinal tracts between the control and overfeeding groups at the phylus level

|  | **Phylum** | **Control (%)** | **Overfeeding (%)** | ***P*-value** |
| --- | --- | --- | --- | --- |
| Jejunum | *Actinobacteria* | 0.0473±2.13×10^-4^ | 8.42±1.27 | 4.52×10^-2^ |
|  | *Fusobacteria* | 2.11×10^-4^±1.75×10^-5^ | 0.0116±1.70×10^-3^ | 4.27×10^-2^ |
|  | *Tenericutes* | 1.33×10^-5^±3.44×10^-6^ | 8.98×10^-4^±1.27×10^-4^ | 2.98×10^-2^ |
|  | *Parcubacteria* | 6.98×10^-6^±1.80×10^-6^ | 8.86×10^-4^±1.32×10^-4^ | 4.12×10^-2^ |
|  | *Moranbacteria* | 1.94×10^-6^±4.99×10^-7^ | 6.80×10^-4^±9.28×10^-5^ | 2.60×10^-2^ |
|  | *Synergistetes* | 0 | 4.24×10^-4^±6.15×10^-5^ | 3.29×10^-2^ |
|  | *Woesebacteria* | 0 | 2.05×10^-4^±3.05×10^-5^ | 3.73×10^-2^ |
|  | *Dictyoglomi* | 0 | 1.16×10^-4^±1.76×10^-5^ | 4.63×10^-2^ |
| Ileum | *Firmicutes* | 17.2±1.36 | 55.2±4.77 | 4.40×10^-2^ |
|  | *Euryarchaeota* | 8.76×10^-3^±1.71×10^-4^ | 2.25×10^-3^±3.79×10^-4^ | 2.93×10^-3^ |
|  | *Zambryskibacteria* | 7.30×10^-5^±1.42×10^-5^ | 3.98×10^-4^±3.62×10^-5^ | 3.08×10^-2^ |
| Cecum | *Firmicutes* | 37.6±0.920 | 9.00±0.916 | 2.95×10^-3^ |
|  | *Bacteroidetes* | 37.3±1.36 | 5.54±0.548 | 2.67×10^-3^ |
|  | *Euryarchaeota* | 1.23±0.0879 | 3.65×10^-3^±2.57×10^-4^ | 1.02×10^-2^ |
|  | *Synergistetes* | 0.719±0.0703 | 5.94×10^-3^±6.12×10^-4^ | 2.45×10^-2^ |
|  | *Elusimicrobia* | 0.381±0.0168 | 1.20×10^-3^±9.19×10^-4^ | 1.42×10^-3^ |
|  | *Chlamydiae* | 0.0354±2.82×10^-3^ | 0.461±0.0283 | 7.97×10^-3^ |
|  | *Mucoromycota* | 7.34×10^-3^±1.02×10^-4^ | 0.0756±6.53×10^-3^ | 1.05×10^-2^ |
|  | *Ascomycota* | 7.3×10^-3^±7.72×10^-4^ | 0.102±6.53×10^-3^ | 9.11×10^-3^ |
|  | *Basidiomycota* | 3.39×10^-3^±3.28×10^-4^ | 0.0479±3.95×10^-3^ | 8.54×10^-3^ |
|  | *Microsporidia* | 9.49×10^-4^±1.30×10^-4^ | 5.20×10^-3^±3.28×10^-4^ | 1.38×10^-2^ |
|  | *Chytridiomycota* | 6.51×10^-4^±8.96×10^-5^ | 0.0618±4.80×10^-3^ | 7.12×10^-3^ |
|  | *Blastocladiomycota* | 1.50×10^-4^±2.45×10^-5^ | 0.0145±3.05×10^-3^ | 6.06×10^-3^ |
|  | *Cryptomycota* | 6.13×10^-5^±1.17×10^-5^ | 8.28×10^-3^±6.12×10^-4^ | 1.15×10^-2^ |

Note: the relative abundance of intestinal bacteria was determined by metagenome analysis. n=4.
