# Supplementary material for: The Changes in Microbiotic Composition of Different Intestinal Tracts and the Effects of Supplemented Lactobacillus During the Formation of Goose Fatty Liver
Source: Front Microbiol. 2022 Jul 18;13:906895. doi: 10.3389/fmicb.2022.906895 (PMC9339986; doi:10.3389/fmicb.2022.906895)
Supplement: Supplementary file 5 [file Table_5.docx]

**Supplementary Table 5.** The relative abundance of the main differential bacteria (≥1%) at the genus level between the control group and the overfeeding group

|  | **Genus** | **Control (%)** | **Overfeeding (%)** | ***P*-value** |
| --- | --- | --- | --- | --- |
| 12d Jejunum | *Helicobacter* | 16.5±2.26 | 32.6±5.81 | 1.21×10^-2^ |
|  | *Lactobacillus* | 2.35±0.479 | 5.92±0.919 | 1.28×10^-3^ |
|  | *Streptococcus* | 1.31±0.323 | 5.22±1.25 | 3.83×10^-3^ |
|  | *Unidentified_Chloroplast* | 7.19±3.07 | 0.870±0.366 | 4.35×10^-2^ |
|  | *Romboutsia* | 6.31±1.33 | 2.40±0.425 | 6.93×10^-3^ |
|  | *Tyzzerella_3* | 3.55±1.31 | 0.0677±0.0510 | 1.02×10^-2^ |
|  | *Lactococcus* | 1.86±0.381 | 0.809±0.305 | 3.44×10^-2^ |
|  | *Clostridium*  *_sensu_stricto_1* | 1.48±0.201 | 0.419±0.146 | 1.16×10^-4^ |
| 12d Ileum | *Phyllobacterium* | 18.6±2.59 | 31.2±4.62 | 2.29×10^-2^ |
|  | *Lactobacillus* | 2.18±0.510 | 9.74±2.73 | 1.08×10^-2^ |
|  | *Enterococcus* | 3.32±0.402 | 7.77±1.39 | 4.52×10^-3^ |
|  | *Streptococcus* | 1.92±0.439 | 5.63±1.32 | 1.20×10^-2^ |
|  | *Weissella* | 1.28±0.176 | 4.07±0.554 | 4.43×10^-5^ |
|  | *Helicobacter* | 11.8±2.27 | 5.63±1.26 | 2.05×10^-2^ |
|  | *Romboutsia* | 11.3±2.23 | 3.80±1.72 | 1.13×10^-2^ |
|  | *Tyzzerella_3* | 3.47±1.45 | 0.177±0.109 | 2.58×10^-2^ |
|  | *Lactococcus* | 2.04±0.431 | 0.528±0.0929 | 1.38×10^-3^ |
|  | *Desulfovibrio* | 1.92±0.214 | 1.05±0.229 | 8.26×10^-3^ |
| 12d Cecum | *Phyllobacterium* | 3.86±0.737 | 7.61±1.52 | 2.84×10^-2^ |
|  | *Enterococcus* | 1.20±0.365 | 3.63±0.577 | 9.25×10^-4^ |
|  | *Lactobacillus* | 0.895±0.580 | 3.43±0.637 | 4.95×10^-3^ |
|  | *Streptococcus* | 0.652±0.221 | 2.53±0.495 | 1.17×10^-3^ |
| 24d Jejunum | *Lactobacillus* | 8.90±4.02 | 26.0±4.22 | 5.00×10^-3^ |
|  | *Enterococcus* | 3.78±0.792 | 19.3±2.35 | 3.75×10^-7^ |
|  | *Weissella* | 1.92±0.477 | 6.81±2.11 | 2.60×10^-2^ |
|  | *Bacteroides* | 0.961±0.301 | 4.51±1.34 | 1.21×10^-2^ |
|  | *Veillonella* | 0.709±0.110 | 4.29±0.960 | 6.18×10^-4^ |
|  | *Phyllobacterium* | 45.3±4.69 | 0.387±0.169 | 6.08×10^-11^ |
| 24d Ileum | *Enterococcus* | 3.98±0.713 | 18.6±2.89 | 1.87×10^-5^ |
|  | *Lactobacillus* | 3.86±0.579 | 18.1±3.67 | 4.32×10^-4^ |
|  | *Gallibacterium* | 0.175±0.134 | 1.41±0.373 | 3.20×10^-3^ |
|  | *Phyllobacterium* | 43.2±4.74 | 0.167±0.0700 | 2.04×10^-10^ |
|  | *Romboutsia* | 6.03±1.91 | 0.546±0.369 | 6.67×10^-3^ |
|  | *Turicibacter* | 1.74±0.774 | 0.0898±0.0535 | 3.58×10^-2^ |
|  | *Desulfovibrio* | 1.60±0.329 | 0.726±0.214 | 2.85×10^-2^ |
| 24d Cecum | *Enterococcus* | 3.25±1.74 | 8.70±1.44 | 1.84×10^-2^ |
|  | *Lactobacillus* | 3.01±0.904 | 8.59±1.88 | 9.82×10^-3^ |
|  | *Escherichia-Shigella* | 0.521±0.170 | 26.5±5.46 | 3.03×10^-5^ |
|  | *Veillonella* | 0.457±0.152 | 1.14±0.201 | 9.08×10^-3^ |
|  | *Gallibacterium* | 0.215±0.119 | 1.80±0.422 | 7.81×10^-4^ |
|  | *Bacteroides* | 22.4±1.31 | 12.8±3.86 | 2.17×10^-2^ |
|  | *Phyllobacterium* | 6.47±1.28 | 0.0255±7.18×10^-3^ | 1.30×10^-5^ |
|  | *Rikenellaceae_RC9* | 3.06±0.517 | 0.159±0.108 | 1.48×10^-5^ |
|  | *Ruminococcaceae_UCG-014* | 2.60±0.249 | 0.272±0.195 | 1.77×10^-8^ |
|  | *Lachnoclostridium* | 1.72±0.170 | 0.616±0.128 | 8.39×10^-6^ |

Note: the relative abundance of intestinal bacteria was determined by 16S rRNA analysis. n=16.
