# Supplementary material for: The Changes in Microbiotic Composition of Different Intestinal Tracts and the Effects of Supplemented Lactobacillus During the Formation of Goose Fatty Liver
Source: Front Microbiol. 2022 Jul 18;13:906895. doi: 10.3389/fmicb.2022.906895 (PMC9339986; doi:10.3389/fmicb.2022.906895)
Supplement: Supplementary file 6 [file Table_6.docx]

**Supplementary Table 6.** The relative abundance of differential bacteria in different intestinal tracts between the control and overfeeding groups at the genus level

|  | **Genus** | **Control (%)** | **Overfeeding (%)** | ***P*-value** |
| --- | --- | --- | --- | --- |
| Jejunum | *Avipoxvirus* | 6.51×10^-5^±1.04×10^-5^ | 3.43×10^-5^±1.41×10^-5^ | 2.77×10^-2^ |
|  | *Proboscivirus* | 3.23×10^-5^±6.72×10^-6^ | 1.47×10^-5^±6.13×10^-6^ | 1.92×10^-2^ |
|  | *Tilletia* | 2.19×10^-5^±6.07×10^-6^ | 9.07×10^-6^±4.55×10^-6^ | 5.00×10^-2^ |
|  | *Calocera* | 1.67×10^-5^±3.06×10^-6^ | 7.78×10^-6^±2.40×10^-6^ | 9.62×10^-3^ |
|  | *Lymphocystivirus* | 1.44×10^-5^±3.07×10^-6^ | 5.86×10^-6^±2.81×10^-6^ | 1.49×10^-2^ |
|  | *Streptococcus* | 7.78×10^-5^±3.54×10^-5^ | 0.0268±0.0155 | 3.78×10^-2^ |
|  | *Rothia* | 2.84×10^-5^±2.42×10^-5^ | 0.0145±8.39×10^-3^ | 3.41×10^-2^ |
|  | *Staphylococcus* | 0.000135±5.05×10^-5^ | 2.96×10^-3^±1.64×10^-3^ | 4.09×10^-2^ |
|  | *Tessaracoccus* | 7.52×10^-6^±3.64×10^-6^ | 1.58×10^-3^±8.13×10^-4^ | 1.92×10^-2^ |
|  | *Arthrobacter* | 2.05×10^-6^±1.08×10^-6^ | 6.91×10^-4^±3.96×10^-3^ | 2.98×10^-2^ |
| Ileum | *Sulfurospirillum* | 1.18×10^-4^±6.68×10^-5^ | 2.59×10^-6^±1.93×10^-6^ | 4.86×10^-2^ |
|  | *Wolinella* | 3.59×10^-5^±1.93×10^-5^ | 3.45×10^-7^±3.45×10^-7^ | 3.57×10^-2^ |
|  | *Firmicutes* | 3.19×10^-5^±1.06×10^-5^ | 3.45×10^-7^±3.45×10^-7^ | 5.24×10^-3^ |
|  | *Kurthia* | 2.10×10^-5^±1.17×10^-5^ | 5.32×10^-7^±3.52×10^-7^ | 4.41×10^-2^ |
|  | *Cellulosilyticum* | 1.75×10^-5^±8.79×10^-6^ | 2.50×10^-7^±2.50×10^-7^ | 2.79×10^-2^ |
|  | *Lactobacillus* | 0.0481±0.0312 | 0.227±0.0898 | 3.33×10^-2^ |
|  | *Leptotrichia* | 9.19×10^-6^±3.82×10^-6^ | 2.76×10^-4^±1.50×10^-4^ | 4.55×10^-2^ |
|  | *Tessaracoccus* | 2.68×10^-5^±9.55×10^-6^ | 2.92×10^-4^±1.51×10^-4^ | 4.34×10^-2^ |
|  | *Holdemania* | 1.98×10^-6^±2.32×10^-7^ | 1.56×10^-4^±8.75×10^-5^ | 4.29×10^-2^ |
|  | *Dehalobacter* | 2.79×10^-5^±1.91×10^-5^ | 1.76×10^-4^±6.31×10^-5^ | 1.71×10^-2^ |
| Cecum | *Bacteroides* | 0.211±0.0316 | 0.0413±0.0155 | 3.82×10^-3^ |
|  | *Clostridium* | 0.0634±2.92×10^-3^ | 4.91×10^-3^±2.13×10^-3^ | 1.09×10^-4^ |
|  | *Alistipes* | 0.0483±0.0165 | 2.82×10^-3^±1.77×10^-3^ | 1.84×10^-2^ |
|  | *Desulfovibrio* | 0.0425±0.0120 | 6.82×10^-3^±5.35×10^-3^ | 1.90×10^-2^ |
|  | *Prevotella* | 0.0202±7.56×10^-3^ | 8.19×10^-4^±3.11×10^-4^ | 2.30×10^-2^ |
|  | *Escherichia* | 0.427±0.0251 | 0.851±0.0405 | 8.07×10^-4^ |
|  | *Gammaretrovirus* | 2.68×10^-4^±1.06×10^-4^ | 0.0319±0.0130 | 2.88×10^-2^ |
|  | *Anaplasma* | 3.46×10^-4^±1.09×10^-4^ | 4.60×10^-3^±1.09×10^-3^ | 6.95×10^-3^ |
|  | *Chlamydia* | 5.16×10^-5^±4.89×10^-5^ | 5.87×10^-3^±1.54×10^-3^ | 7.55×10^-3^ |
|  | *Tamlana* | 1.47×10^-5^±1.38×10^-5^ | 1.46×10^-3^±3.52×10^-4^ | 6.01×10^-3^ |

Note: the relative abundance of intestinal bacteria was determined by metagenome analysis. n=4.
