# Supplementary material for: The Changes in Microbiotic Composition of Different Intestinal Tracts and the Effects of Supplemented Lactobacillus During the Formation of Goose Fatty Liver
Source: Front Microbiol. 2022 Jul 18;13:906895. doi: 10.3389/fmicb.2022.906895 (PMC9339986; doi:10.3389/fmicb.2022.906895)
Supplement: Supplementary file 7 [file Table_7.docx]

**Supplementary Table 7.** The differential KEGG pathways at the secondary tier based on the relative abundance of bacterial Unigenes in different intestinal tracts

|  | **Pathway** | **Control（%）** | **Overfeeding（%）** | ***P*-value** |
| --- | --- | --- | --- | --- |
| Ileum | Environmental Information Processing Signal Transduction | 8.41×10^-3^±1.02×10^-3^ | 0.0137±1.45×10^-3^ | 3.27×10^-2^ |
|  | Cellular Processes Cellular Community | 5.05×10^-3^±5.83×10^-4^ | 0.0111±2.20×10^-3^ | 4.92×10^-2^ |
|  | Organismal Systems Endocrine System | 2.40×10^-3^±2.14×10^-4^ | 3.94×10^-3^±1.07×10^-4^ | 1.19×10^-3^ |
| Cecum | Metabolism Global and Overview Maps | 0.0366±9.61×10^-4^ | 0.0127±5.11×10^-3^ | 1.27×10^-2^ |
|  | Metabolism Carbohydrate Metabolism | 0.0361±7.07×10^-4^ | 0.0155±6.21×10^-3^ | 1.55×10^-2^ |
|  | Metabolism Amino Acid Metabolism | 0.0305±7.80×10^-4^ | 0.0118±4.70×10^-3^ | 1.18×10^-2^ |
|  | Genetic Information Processing Translation | 0.0242±4.23×10^-4^ | 6.89×10^-3^±2.31×10^-3^ | 6.89×10^-3^ |
|  | Metabolism Energy Metabolism | 0.0227±4.68×10^-4^ | 8.67×10^-3^±3.36×10^-3^ | 8.67×10^-3^ |
|  | Metabolism Metabolism of Cofactors and Vitamins | 0.0219±2.47×10^-4^ | 8.67×10^-3^±3.51×10^-3^ | 8.67×10^-3^ |
|  | Metabolism Nucleotide Metabolism | 0.0211±4.12×10^-4^ | 7.14×10^-3^±2.70×10^-3^ | 7.14×10^-3^ |
|  | Genetic Information Processing Replication and Repair | 0.0142±2.53×10^-4^ | 4.30×10^-3^±1.62×10^-3^ | 4.30×10^-3^ |
|  | Metabolism Glycan Biosynthesis and Metabolism | 0.0106±4.61×10^-4^ | 3.96×10^-3^±1.34×10^-3^ | 3.96×10^-3^ |
|  | Genetic Information Processing Folding_ Sorting and Degradation | 9.74×10^-3^±1.57×10^-4^ | 3.83×10^-3^±1.21×10^-3^ | 3.83×10^-3^ |
|  | Metabolism Lipid Metabolism | 8.20×10^-3^±4.08×10^-5^ | 3.64×10^-3^±1.27×10^-3^ | 3.64×10^-3^ |
|  | Metabolism of Other Amino Acids | 7.41×10^-3^±1.09×10^-4^ | 3.37×10^-3^±1.35×10^-3^ | 3.37×10^-3^ |
|  | Human Diseases Antimicrobial Resistance | 5.92×10^-3^±1.94×10^-4^ | 2.47×10^-3^±1.12×10^-3^ | 2.47×10^-3^ |
|  | Metabolism Biosynthesis of Other Secondary Metabolites | 5.86×10^-3^±1.14×10^-4^ | 1.81×10^-3^±7.11×10^-4^ | 1.81×10^-3^ |
|  | Metabolism Metabolism of terpenoids and Polyketides | 5.29×10^-3^±3.60×10^-5^ | 2.06×10^-3^±8.06×10^-4^ | 2.06×10^-3^ |
|  | Cellular Processes Cell growth and Death | 3.60×10^-3^±5.56×10^-5^ | 1.50×10^-3^±2.42×10^-4^ | 1.50×10^-3^ |
|  | Cellular Processes Transport and Catabolism | 2.15×10^-3^±1.74×10^-4^ | 1.60×10^-3^±1.32×10^-4^ | 1.60×10^-3^ |
|  | Organismal Systems Aging | 1.70×10^-3^±4.96×10^-5^ | 8.06×10^-4^±9.53×10^-5^ | 8.06×10^-4^ |
|  | Genetic Information Processing Transcription | 1.65×10^-3^±6.55×10^-5^ | 1.19×10^-3^±1.07×10^-4^ | 1.19×10^-3^ |
|  | Human Diseases Endocrine and Metabolic Diseases | 1.54×10^-3^±3.57×10^-5^ | 2.04×10^-3^±1.77×10^-4^ | 2.04×10^-3^ |
|  | Cellular Processes Cell Motility | 1.48×10^-3^±0.0002.70×10^-4^ | 3.15×10^-3^±2.96×10^-4^ | 3.15×10^-3^ |
|  | Organismal Systems Environmental Adaptation | 8.26×10^-4^±2.42×10^-5^ | 6.83×10^-4^±4.82×10^-5^ | 6.83×10^-4^ |
|  | Organismal Systems Nervous System | 7.42×10^-4^±4.28×10^-5^ | 2.39×10^-3^±4.04×10^-4^ | 2.39×10^-3^ |
|  | Human Diseases Neurodegenerative Diseases | 5.85×10^-4^±1.05×10^-5^ | 8.52×10^-4^±7.23×10^-5^ | 8.52×10^-4^ |
|  | Human Diseases Cancers Specific Types | 3.82×10^-4^±1.42×10^-5^ | 1.99×10^-3^±3.69×10^-4^ | 1.99×10^-3^ |
|  | Organismal Systems Immune System | 3.51×10^-4^±1.31×10^-5^ | 2.10×10^-3^±4.30×10^-4^ | 2.10×10^-3^ |
|  | Organismal Systems Digestive System | 3.29×10^-4^±4.16×10^-5^ | 1.17×10^-3^±2.20×10^-4^ | 1.16×10^-3^ |
|  | Human Diseases Antineoplastic Resistance | 1.84×10^-4^±1.90×10^-5^ | 3.08×10^-4^±2.00×10^-5^ | 3.08×10^-4^ |
|  | Organismal Systems Excretory System | 0.0001.39×10^-4^±1.57×10^-5^ | 6.16×10^-4^±1.11×10^-4^ | 6.16×10^-4^ |
|  | Human Diseases Infectious Diseases Viral | 1.08×10^-4^±3.54×10^-6^ | 1.30×10^-3^±3.13×10^-4^ | 1.30×10^-3^ |
|  | Environmental Information Processing Signaling Molecules and Interaction | 6.51×10^-5^±8.89×10^-6^ | 1.05×10^-3^±2.50×10^-4^ | 1.05×10^-3^ |
|  | Human Diseases Infectious Diseases Parasitic | 6.40×10^-5^±9.51×10^-6^ | 3.79×10^-4^±7.07×10^-5^ | 3.79×10^-4^ |
|  | Organismal Systems Sensory System | 4.18×10^-5^±3.78×10^-5^ | 3.71×10^-3^±9.64×10^-4^ | 3.71×10^-3^ |
|  | Human Diseases Cardiovascular Diseases | 1.94×10^-5^±5.57×10^-6^ | 4.80×10^-4^±1.18×10^-4^ | 4.80×10^-4^ |
|  | Organismal Systems Development | 1.24×10^-5^±1.13×10^-5^ | 1.47×10^-3^±3.25×10^-4^ | 1.47×10^-3^ |
|  | Organismal Systems Circulatory System | 7.10×10^-6^±4.33×10^-6^ | 6.07×10^-4^±1.55×10^-4^ | 6.07×10^-4^ |
|  | Human Diseases Substance Dependence | 4.22×10^-6^±2.69×10^-6^ | 5.36×10^-4^±1.33×10^-4^ | 5.36×10^-4^ |

Note: The differential pathways were identified based on the differences in the relative abundance of bacterial Unigenes between the overfeeding and control groups. The relative abundance of bacterial Unigenes was determined by metagenome analysis on intestinal bacteria.
