# Supplementary material for: The Changes in Microbiotic Composition of Different Intestinal Tracts and the Effects of Supplemented Lactobacillus During the Formation of Goose Fatty Liver
Source: Front Microbiol. 2022 Jul 18;13:906895. doi: 10.3389/fmicb.2022.906895 (PMC9339986; doi:10.3389/fmicb.2022.906895)
Supplement: Supplementary file 8 [file Table_8.docx]

**Supplementary Table 8.** The top 10 differential KEGG pathways at the third tier based on the relative abundance of bacterial Unigenes in different intestinal tracts

|  | **Pathway** | **Control（%）** | **Overfeeding（%）** | ***P*-value** |
| --- | --- | --- | --- | --- |
| Jejunum | African Trypanosomiasis | 3.60×10^-5^±5.03×10^-6^ | 6.88×10^-5^±1.27×10^-5^ | 3.54×10^-2^ |
| Ileum | Phosphatidylinositol Signaling System | 3.23×10^-4^±2.89×10^-5^ | 4.93×10^-4^±6.21×10^-5^ | 4.98×10^-2^ |
|  | RNA Transport | 2.87×10^-4^±4.84×10^-5^ | 4.73×10^-4^±3.99×10^-5^ | 2.62×10^-2^ |
|  | Thyroid Hormone Synthesis | 2.27×10^-4^±1.22×10^-5^ | 3.63×10^-4^±3.34×10^-5^ | 7.73×10^-3^ |
|  | PPAR Signaling Pathway | 1.98×10^-4^±2.41×10^-5^ | 4.76×10^-4^±1.02×10^-4^ | 3.95×10^-2^ |
|  | NOD-like Receptor Signaling Pathway | 8.45×10^-5^±1.96×10^-5^ | 2.53×10^-5^±7.92×10^-6^ | 3.33×10^-2^ |
|  | Cutin, Suberine and Wax Biosynthesis | 2.81×10^-6^±6.84×10^-7^ | 1.46×10^-7^±1.46×10^-7^ | 8.05×10^-3^ |
| Cecum | Biosynthesis of Amino Acids | 0.0221±6.66×10^-4^ | 7.79×10^-3^±3.05×10^-3^ | 6.10×10^-3^ |
|  | Carbon Metabolism | 0.0161±4.58×10^-4^ | 5.66×10^-3^±2.22×10^-3^ | 5.90×10^-3^ |
|  | Purine Metabolism | 0.0137±2.33×10^-4^ | 4.77×10^-3^±1.74×10^-3^ | 5.13×10^-3^ |
|  | Ribosome | 0.0132±1.53×10^-4^ | 3.90×10^-3^±1.46×10^-3^ | 2.04×10^-3^ |
|  | Pyrimidine Metabolism | 0.0116±2.37×10^-4^ | 3.80×10^-3^±1.44×10^-3^ | 4.31×10^-3^ |
|  | Amino Sugar and Nucleotide Sugar Metabolism | 7.56×10^-3^±7.32×10^-5^ | 2.56×10^-3^±9.43×10^-4^ | 4.45×10^-3^ |
|  | Glycolysis / Gluconeogenesis | 7.28×10^-3^±1.82×10^-4^ | 2.33×10^-3^±8.48×10^-4^ | 3.15×10^-3^ |
|  | Pyruvate Metabolism | 6.34×10^-3^±1.96×10^-4^ | 2.31×10^-3^±9.44×10^-4^ | 7.39×10^-3^ |
|  | Oxidative Phosphorylation | 5.31×10^-3^±5.30×10^-5^ | 2.37×10^-3^±8.40×10^-4^ | 1.83×10^-2^ |
|  | Olfactory Transduction | 3.56×10^-5^±3.30×10^-5^ | 3.24×10^-3^±8.58×10^-4^ | 1.53×10^-2^ |

Note: The differential pathways were identified based on the differences in the relative abundance of bacterial Unigenes between the overfeeding and control groups. The relative abundance of bacterial Unigenes was determined by metagenome analysis on intestinal bacteria.
