# Supplementary figures and images for: The Changes in Microbiotic Composition of Different Intestinal Tracts and the Effects of Supplemented Lactobacillus During the Formation of Goose Fatty Liver
Source: Front Microbiol. 2022 Jul 18;13:906895. doi: 10.3389/fmicb.2022.906895 (PMC9339986; doi:10.3389/fmicb.2022.906895)

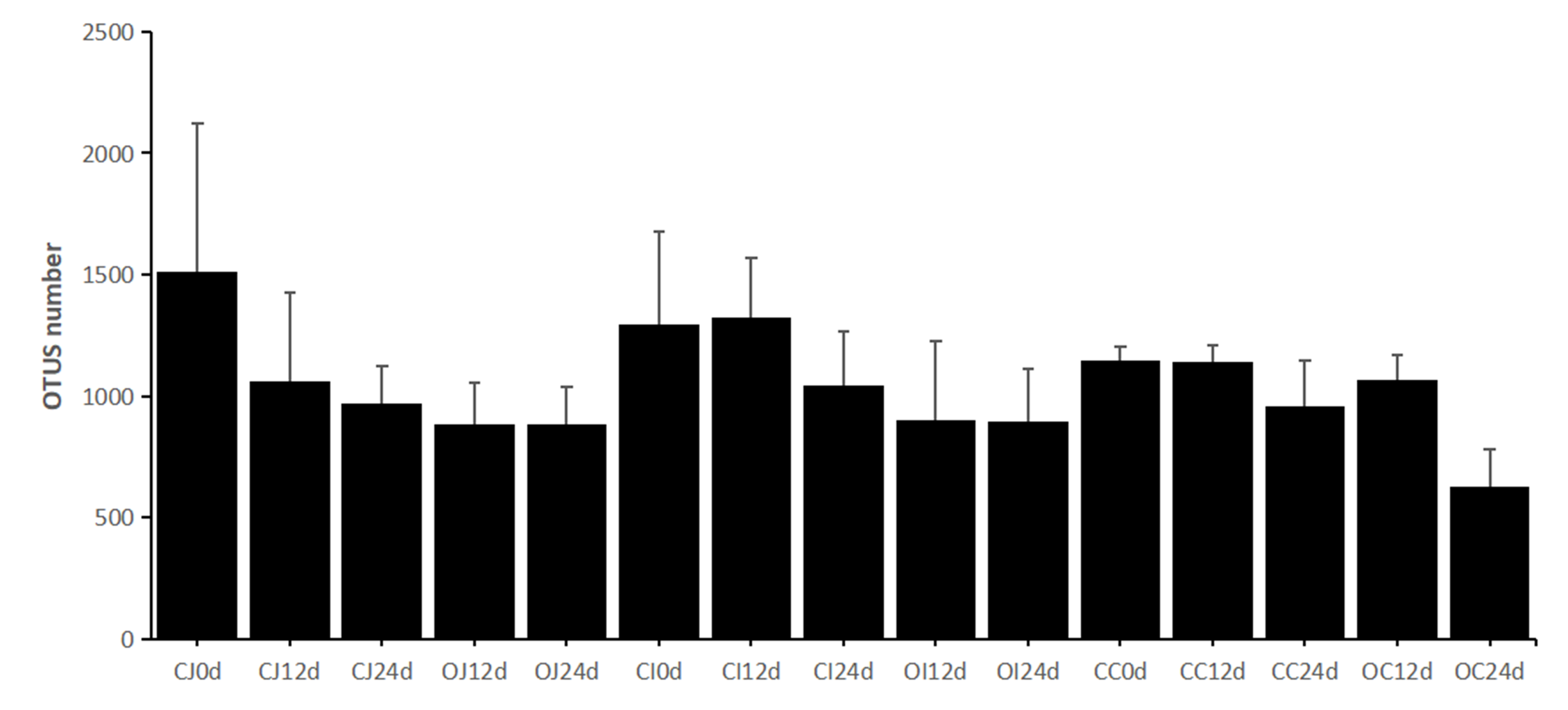

Supplement: Supplementary file 9 [file Image_1.tif]

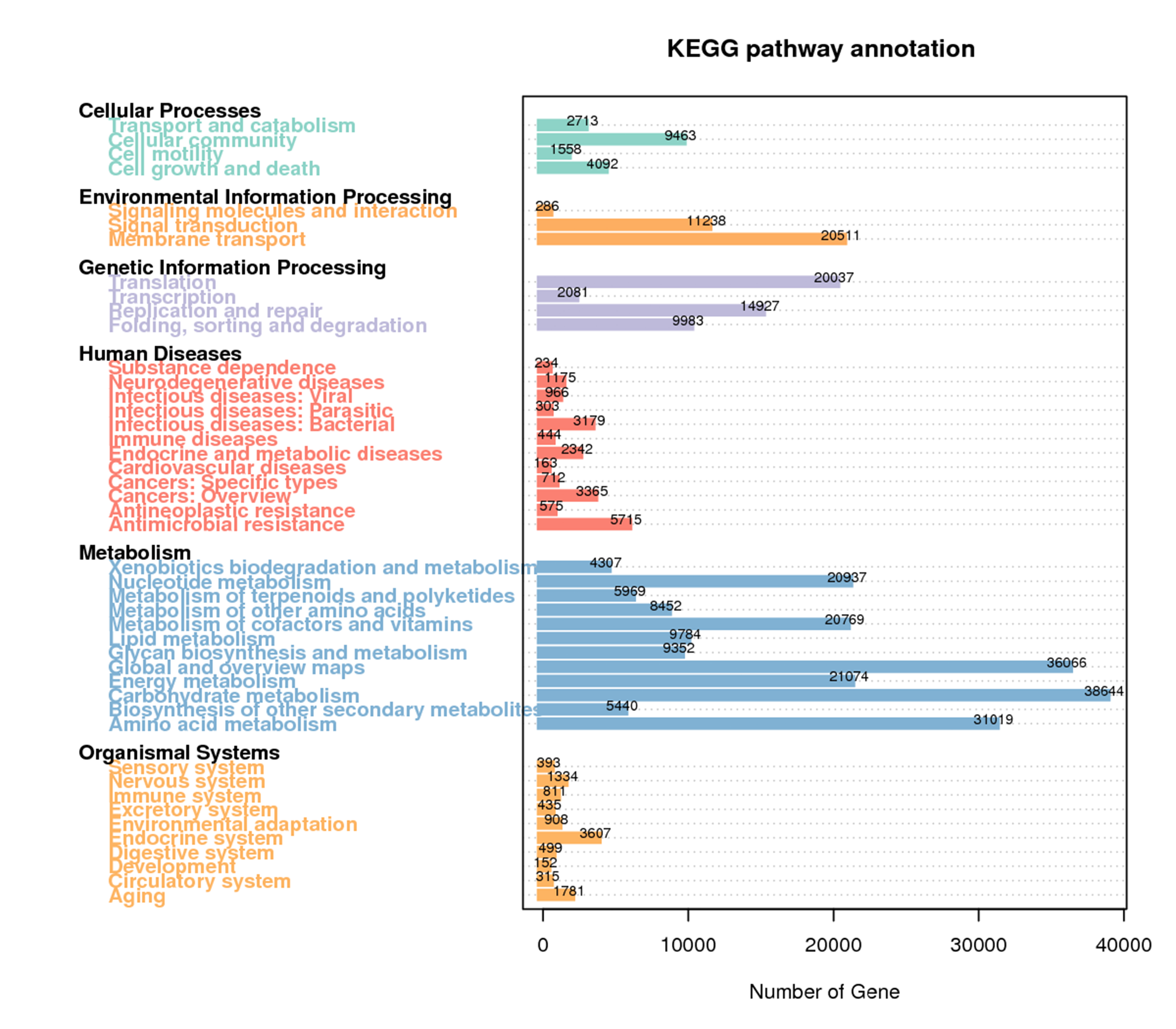

Supplement: Supplementary file 10 [file Image_2.tif]
